# Minimizing Lundberg inequality for ruin probability under correlated risk model by investment and reinsurance

**DOI:** 10.1186/s13660-018-1838-0

**Published:** 2018-09-17

**Authors:** Lin Xu, Minghan Wang, Bin Zhang

**Affiliations:** School of Mathematics and Statistics, Anhui Normal University, Wuhu, China

**Keywords:** Lundberg inequality, Ruin probability, Optimal reinsurance, Optimal investment, Correlated risk model

## Abstract

This paper investigates optimal investment and reinsurance policies for an insurance company under a correlated risk model with common Poisson shocks. The goal of the insurance company is to minimize the ultimate ruin probability. By the dynamic programming principle, the Hamilton–Jacobi–Bellman (HJB for short) equation associated with this control problem is obtained. Since there is no explicit solution to the HJB equation, this paper alternates to find the minimal exponential upper bound of the ruin probability. The exponential upper bound of ruin probability is also called Lundberg inequality. Minimizing Lundberg inequality is equal to finding the maximal Lundberg coefficient. It turns out that the optimal investment and reinsurance polices are constant policies. Some numerical examples are given to illustrate the impact of the dependent structure and the investment chance on the upper bound.

## Introduction

The past two decades have witnessed huge attention on the risk model with dependent structure. The dependent structure is variety. For example, Cossette and Marceau [1], Yuen *et al.* [2] studied ruin probability under a model with dependent business; Wang and Yuen [3] studied ruin probability when the premiums and claims are thinning dependent; Wang and Yin [4] studied asymptotic ruin probabilities in a dependent discrete risk model. On the other hand, there is also a large amount of literature focus on the model when the claims and claim arrival intervals are correlated; for example, see Denuit *et al.* [5], Boudreault *et al.* [6] and the references therein. There is also a lot of literature concentrating on a multivariate risk process, where the components of the multivariate process specify different business of insurance company and they cannot be integrated into a univariate process (or it is meaningless in practice). For details on this aspect, see Weng *et al.* [7], Anastasiadis and Chukova [8] and the references therein.

Almost every insurance company has investment and reinsurance plans for profit increments and risk exposure control. Due to this fact and regulatory requirements, research on optimal reinsurance and (or) investment for an insurance company has become one of the most important topics in risk theory recently (cf. Browne [9], Fleming and Sheu [10], Taksar and Markussen [11], Yang and Zhang [12], Zhang and Siu [13], Zeng and Li [14], Meng *et al.* [15]). The aforemention papers did not consider dependent structure or correlated structure. Naturally, it makes sense to consider the optimal investment and reinsurance problem under a correlated risk model. [16] studied optimal dynamic reinsurance with dependent risks and variance premium principle, where the goal of insurance company is to maximize exponential utility at terminal time. Liang and Yuen [17] investigated optimal investment and reinsurance strategies for an insurance company with generalized mean-variance premium principle and no-short selling, the goal of the insurance company wherein is to maximize the expected utility. Other works on this topic can also be found in Landriault *et al*. [18], Zeng *et al.* [19], Chiu and Wong [20].

Compared to the study on optimal investment and reinsurance for maximizing expected utility, papers concentrating on minimizing ultimate ruin probability are relatively few. Usually, if one wants to find optimal policies for minimizing the ultimate ruin probability, it is difficult to prove the regularity of the value function. It is also very difficult to obtain an explicit solution to the HJB equation and the optimal policies. This may be one of the reasons that prevent us from progressing the study on minimizing ultimate ruin probability by investment or reinsurance. When the explicit expression of ruin probability is invalid, it is meaningful to find an accurate upper bound estimation of ruin probability. Due to this fact, the upper bound estimation of ruin probability has become one of the three main topics in the classical risk model (cf. Grandell [21]). In the classical risk model, an upper bound for ruin probability is also called Lundberg inequality. Hu and Zhang [22] studied optimal reinsurance for minimizing the upper bound of ultimate ruin probability in a correlated risk model with common shocks. The results in [22] show that the upper bound of the ruin probability in the model with reinsurance chance is less than the one in the model without reinsurance chance. Thus, under the correlated risk model, reinsurance business can really reduce the exposure of the insurance company, and this impact can be quantified by comparing the upper bound. Hu and Zhang [22] only considered reinsurance business, it is natural to take both reinsurance and investment into account. It is well known that if the claim sizes have exponential moments (i.e., the so-called small claim case), the ruin probability decreases exponentially with the increase in the initial surplus (cf. Assussen and Albrecher [23]). However, Kalashnikov and Norberg [24], Frolova *et al*. [25] found that, even if the claim sizes are small, if the insurance company invests all of its surplus into a risky market, the ruin probability decreases only with some negative power function of the initial surplus. Thus, for large capital, investing more than the surplus into the risky market cannot be optimal. Then, one interesting problem is: What is the minimal ruin probability that it can obtain? Particularly, can it do better than keeping the funds in the bond? And if yes, how much can it do better? Hipp and Plum [26] and Gaier *et al.* [27] considered this problem when the surplus process is the compound Poisson process and found that when the insurance company can adjust its investment amount to reduce the risk exposure, a constant investment amount is the optimal policy, regardless of the change of surplus of the insurance company. This paper extends the study of [22] by incorporating the investment business into the decision process. But the method applied in this paper relies on the dynamic programming principle and HJB equation. As a result, we also find that the optimal polices for minimizing the upper bound of ruin probability are constant investment amount and constant reinsurance ratio, respectively. Numerical examples show the following: under optimal constant investment policy, an upper bound of the ultimate ruin probability is less than the corresponding one in [22]; when the correlation coefficient of each component risk process increases, the impact of investment on the upper bound of ruin probability inequality is less significant; when the claims are heavy-tailed, reinsurance plays a more important role for the insurance company than the investment.

The rest of this paper is organized as follows. Section 2 presents the model and problem. Section 3 gives the analysis process and the equations satisfying minimal adjustment coefficients. Section 4 gives comparison numerical examples and our conclusions.

## Model and problem

In this paper, we revisit the correlated risk model with dependent common Poisson shock structure that was brought forward by Hu and Zhang [22]. Assume that an insurance company has *n* ($n \geq2$) dependent business. Suppose that random events that may cause a claim in at least one of the *n* classes are classified into *m* ($m\geq1$) types. Denote by $N^{(k)}(t)$ the number of events from the *k*th type that occurred up to time *t*, which is a Poisson process tensity $\eta_{k}$, $k = 1,\ldots,m$. The processes $N^{(1)}(t),\ldots,N^{(m)}(t)$ are assumed to be independent. Let $N_{i}(t)$ be the claims arrival process of the *i*th business, which can be specified by

1 $$ N_{i}(t)=\sum_{k=1}^{m} \alpha_{ik} \circ N^{(k)}(t), $$

where $\alpha_{ik} \in[0, 1]$ meaning that each event that occurred at time *t* in the *k*th type may cause a claim in the *i*th class with probability $\alpha_{ik}$, for $i = 1,\ldots,n$, and $k = 1,\ldots,m$. The thinning procedure “∘” is denoted by

$$\alpha_{ik} \circ N^{(k)}(t)=\sum _{l=1}^{N^{(k)}(t)}\delta_{ik,l}, $$

where, for any given *i*, *k*, $i = 1,\ldots,n$, $k = 1,\ldots,m$, $\{ \delta_{ik,l};l=1,\ldots,N^{(k)}(t) \}$ are i.i.d. random variables following Bernoulli distribution $B(1,\alpha_{ik})$ and are independent of $N^{(k)}(t)$. Then, for $i = 1,\ldots,n$, $N_{i}(t)$ is a Poisson process with tensity $\lambda_{i}=\sum_{k=1}^{m}\alpha_{ik}\eta_{k}$. The aggregate claims of *i*th class up to time *t* are given by

$$S_{i}(t)=\sum_{j=1}^{N_{i}(t)}Y_{j}^{(i)}, $$

where $Y_{j}^{(i)}$ is the claim amount of the *j*th claim in the *i*th class, $\{Y_{j}^{(i)};j=1,2,\ldots \}$ is assumed to be a sequence of i.i.d. and nonnegative r.v.’s with distribution function $F_{i}$. Then the total claims of the insurance company up to time t is

2 $$ S(t)=\sum_{i=1}^{n}S_{i}(t)= \sum_{i=1}^{n}\sum _{j=1}^{N_{i}(t)}Y_{j}^{(i)}. $$

As usual, we assume that the *n* sequences $\{ Y_{j}^{(1)};j=1,2,\ldots \},\ldots, \{ Y_{j}^{(n)};j=1,2,\ldots \}$ are mutually independent and are independent of $\{N_{i}(t);t\geq0 \}$ for $i = 1,\ldots,n$. Denote by $\{U(t): t\geq0 \}$ the surplus process of the insurance company, then we have

3 $$\begin{aligned} U(t)=x+Ct-S(t)=x+\sum_{i=1}^{n}C_{i}t- \sum_{i=1}^{n}\sum _{j=1}^{N_{i}(t)}Y_{j}^{(i)}, \end{aligned}$$

where *x* ($x\geq0$) is the initial surplus, *C* is the sum of premium income rate for all classes, $C_{i}$ is the premium income rate of the *i*th class, $i = 1,\ldots,n$.

### Remark 1

$N^{(k)}(t)$, $k=1,2,\ldots,m$, are Poisson processes with intensity $\eta_{k}$, $k = 1,\ldots,m$; $\alpha_{ik} \circ N^{(k)}(t)\sim B(N^{(k)}(t),\alpha_{ik})$; $N_{(i)}(t)$, $i=1,2,\ldots,n$, are Poisson processes with tensity $\lambda_{i}$, $i = 1,\ldots,n$.

Suppose that the insurance company can choose to invest in the financial market or to purchase a reinsurance project. For the *i*th business, the insurance company purchases a combination reinsurance of quato-share with $a_{i}$, for $a_{i}\in[0,1]$ and excess-of-loss with retention level $M_{i}\in[0,\infty)$. Once a claim in the *i*th class occurs, the insurance company pays $\tilde {Y}^{(i)} = \min \{a_{i}Y^{(i)},M_{i} \}$, and the reinsurance company pays $Y^{(i)}-\tilde{Y}^{(i)}= \max \{ (1-a_{i})Y^{(i)},Y^{(i)}-M_{i} \}$. The reinsurance company receives premium rate from the insurance company $C_{i}^{R}$. According to the expected value principle, suppose that security loading factors of $C_{i}$ and $C_{i}^{R}$ are $\theta_{i}$ and $\theta_{i}^{R}$ respectively, then

4 $$ \textstyle\begin{cases} C_{i}=(1+\theta_{i})\lambda_{i}\mu_{i},\\ C_{i}^{R}=(1+\theta_{i}^{R})\lambda_{i} ((1-a_{i}) \mu _{i}+ \int_{M_{i}/a_{i}}^{\infty}(a_{i}y-M_{i})\,\mathrm{d}F_{i}(y) ), \end{cases} $$

where $\mu_{i}=E(Y^{(i)})$, $i = 1,\ldots,n$. A reasonable assumption is that the insurance company cannot reinsure the whole risk of the *i*th class with certain profit, it means that safety loading factors $\theta_{i}<\theta_{i}^{R}$, likewise, $a_{i}\in(0,1]$ and $M_{i}\in (0,\infty)$, for $i = 1,\ldots,n$.

After the reinsurance arrangement, let $\tilde{S}(t)$ be the aggregate claims of the insurance company up to time *t* and $\tilde{Y}_{j}^{(i)}$ be the amount of the *j*th claim in the *i*th class with c.d.f. $\tilde{F}_{i}$ and m.g.f. $\tilde{\phi}_{i}$. For simplicity of notation, let $\mathbf{a}= (a_{1},\ldots,a_{n})$, $\mathbf {M}=(M_{1},\ldots,M_{n})$ and $C^{I}:=\sum_{i=1}^{n}C_{i}^{I}$. Now a surplus process of the insurance company at time *t* can be rewritten as

5 $$\begin{aligned} U_{\mathbf{a},\mathbf{M}}(t) =&x+C^{I}t-\tilde{S}(t)=x+\sum _{i=1}^{n}\bigl(C_{i}-C_{i}^{R} \bigr)t-\sum_{i=1}^{n}\sum _{j=1}^{N_{i}(t)}\tilde{Y}_{j}^{(i)}. \end{aligned}$$

One should note that both $\sum_{i=1}^{n}\sum_{j=1}^{N_{i}(t)} Y_{j}^{(i)} $ and $\sum_{i=1}^{n}\sum_{j=1}^{N_{i}(t)}\tilde{Y}_{j}^{(i)}$ are pure jump processes. Denote by $\tilde{G}(y)$ the distribution of the jump size of process $\sum_{i=1}^{n}\sum_{j=1}^{N_{i}(t)}\tilde{Y}_{j}^{(i)}$. Denote by $\phi_{i}(r)=\mathbb {E}\mathrm {e}^{rY^{(i)}}$, $\tilde{\phi}_{i}(r)=\mathbb {E}\mathrm {e}^{r\tilde{Y}^{(i)}}$, $i=1,2,\ldots,n$, the moment generating function (m.g.f. for short) of $Y^{(i)}$ and $\tilde{Y}^{(i)}$, respectively. Then we have the following lemma (cf. [22]).

### Lemma 2.1

*The m*.*g*.*f*. *of* $S(t)$ *and* $\tilde{S}(t)$ *are*

$$ M_{S(t)}(r)\mathrel{\hat{=}}\mathbb {E}\bigl(\exp\bigl(rS(t)\bigr)\bigr)=\exp \Biggl(t \sum_{k=1}^{m}\eta_{k} \Biggl( \prod_{i=1}^{n}\bigl(\alpha_{ik} \phi _{i}(r)+1-\alpha_{ik}\bigr)-1 \Biggr) \Biggr) $$

*and*

6 $$ M_{\tilde{S}(t)}(r)\mathrel{\hat{=}} \mathbb {E}\bigl(\exp\bigl(r\tilde{S}(t)\bigr) \bigr)=\exp \Biggl(t\sum_{k=1}^{m} \eta_{k} \Biggl(\prod_{i=1}^{n} \bigl(\alpha_{ik} \tilde{\phi}_{i}(r)+1-\alpha_{ik} \bigr)-1 \Biggr) \Biggr). $$

To distinguish the impact of **a**, **M** on the insurance company, let $Y_{i}(a_{i},M_{i}):=C_{i}^{I}- \tilde{S}_{i}(t)/t$ be the net profit per unit of time for the *i*th class of business, then we have

7 $$ \mathbb {E}\bigl(Y_{i}(a_{i},M_{i}) \bigr)=C_{i}^{I}-\lambda_{i} \mathbb {E}\tilde{Y}^{(i)}. $$

In the classical risk model and the model considered in [22], it is assumed that

8 $$ \mathbb {E}\bigl(Y_{i}(a_{i},M_{i}) \bigr)>0,\quad i=1,\ldots,n. $$

Here we revise this net profit condition into

9 $$ \sum_{i=1}^{n}\mathbb {E}\bigl(Y_{i}(a_{i},M_{i}) \bigr)>0. $$

By the large number law of Markov process, it is easy to know that

10 $$ \lim_{t\rightarrow\infty}U_{\mathbf{a},\mathbf {M}}(t)=\infty. $$

### Remark 2

A natural interpretation of condition (8) is that the insurance company charges more than the net premium of the claims due the expected premium principle. In mathematics, condition (8) guarantees the existence of the root to the Lundberg equation when the claims are “small claims” and thus are of great importance in [22]. However, later we will find that once we take the investment into account, condition (8) is not always necessary. This is one difference between our model and the one of [22].

Besides reinsurance business, we assume that the insurance company would like to invest its surplus into the capital market. Here, we adopt the classical Black–Scholes framework of financial market. Let $r(t)$ and $B(t)$ denote the price of the risk-free asset and risky asset at time *t*, respectively. The dynamics of $r(t)$ and $B(t)$ are

11 $$\begin{aligned}& \mathrm{d}r(t)=r(t)\delta\,\mathrm{d}t, \end{aligned}$$

12 $$\begin{aligned}& \mathrm{d}B(t)=B(t) \bigl(\mu\,\mathrm{d}t+\sigma\,\mathrm{d}W(t)\bigr), \end{aligned}$$

where $W(t)$ is the standard Brownian motion, *δ*, *μ*, and *σ* are positive constants, which denote the constant interest force of risk-free market, the return, and the volatility of financial market, respectively. Denote by $A(t)$ the amount invested into the risky market, by $X_{\mathbf{a},\mathbf{M},A}(t)$ the wealth of the insurance company at time *t* with policy **a**, **M**, *A*. Here we assume that the insurance company can invest the amount more than its current wealth into the risky market, this is a kind of short-run strategy. Now, the wealth process of the insurance company evolves as follows:

13 $$\begin{aligned}& \mathrm{d}X_{\mathbf{a},\mathbf {M},A}(t) \\& \quad =A(t)\frac{\mathrm{d}B(t)}{B(t)}+ \bigl(X_{\mathbf{a},\mathbf {M},A}(t)-A(t) \bigr) \frac{\mathrm{d}r(t)}{r(t)}+\mathrm{d}U_{\mathbf{a},\mathbf{M},A}(t) \\& \quad = A(t) \bigl(\mu\,\mathrm{d}t+\sigma\,\mathrm{d}W(t)\bigr)+ \bigl(X_{\mathbf {a},\mathbf{M},A}(t)-A(t) \bigr)\delta\,\mathrm{d}t+C^{I}\,\mathrm{d}t-\mathrm{d} \Biggl(\sum _{i=1}^{n}\sum_{j=1}^{N_{i}(t)} \tilde{Y}_{j}^{(i)} \Biggr). \end{aligned}$$

Let

$$\begin{aligned} \mathcal{F}_{t} =&\sigma \bigl\{ \bigl(Y_{k}^{1},Y_{k}^{2}, \ldots,Y_{k}^{n}\bigr),k\geq1 \bigr\} \vee\sigma \bigl\{ \bigl(N_{t}^{(1)},N_{t}^{(2)}, \ldots,N_{t}^{(m)}\bigr),0\leq s\leq t \bigr\} \\ &\vee\sigma \bigl\{ W(t),0\leq s\leq t \bigr\} . \end{aligned}$$

### Assumption 1

(i) $0\leq\delta<\mu$, $\mathbf{0}< \mathbf{a}\leq\mathbf {1}$, $\mathbf{0}< \mathbf{M}$, where **0** and **1** are vectors with all the elements being 0 and 1, respectively.
(ii) The investment process $\{A(t)\}_{t\geq0}$ is predictable w.r.t. to $\mathcal{F}_{t}$ and $\mathbb {E}[\int_{0}^{t}|A(s)|^{2}\,\mathrm{d}s ]<\infty$, $\forall t\in[0,\infty)$. This means that the amount of the reinsurance and investment may depend on the information up to time *t* of the system, but it may not depend on the amount of the claims happened at time *t*, i.e., $A(t)\in\mathcal{F}_{t-}$.

Denote by $\mathcal{A}$ the decision policies satisfying Assumption 1. Denote by $\tau_{\mathbf{a},\mathbf{M},A}$ the ruin time of the insurance company with control policies $\{(\mathbf{a},\mathbf {M},A(t)), t\geq0\}$, i.e.,

14 $$ \tau_{\mathbf{a},\mathbf{M},A}(x)=\inf\Bigl\{ t: t\geq 0,\inf_{0\leq s\leq t}X_{\mathbf{a},\mathbf{M},A}(s)< 0,X(0)=x \Bigr\} , $$

by $\psi(x;\mathbf{a},\mathbf{M},A)$ the ruin probability, i.e.,

15 $$ \psi(x;\mathbf{a},\mathbf{M},A)=\mathbb {P}\bigl(\tau _{\mathbf{a},\mathbf{M},A}(x)< \infty \bigr), $$

and by $V(x)$ the value function of the insurance company, i.e.,

16 $$ V(x)=\inf_{(\mathbf{a},\mathbf{M},A)\in\mathcal {A}}\psi(x;\mathbf{a},\mathbf{M},A). $$

Note that the wealth process specified by Eq. (13) is a Markov process with infinitesimal operator

$$\begin{aligned} \mathcal{L}_{\mathbf{a},\mathbf{M},A}f(x) =& \bigl[A(t)\mu+\bigl(x-A(t)\bigr) \delta+C^{I} \bigr]f_{x}+\frac{1}{2}A(t)^{2} \sigma ^{2}f_{xx} \\ &{}+ \int_{0}^{\infty}\bigl[f(x-y)-f(x)\bigr]\,\mathrm{d} \tilde{G}(y), \end{aligned}$$

where $f_{x}$, $f_{xx}$ are the first and second derivatives of *f* w.r.t. *x*. The HJB equation associated with this control problem is

17 $$\begin{aligned}& \inf_{(\mathbf{a},\mathbf{M},A)\in\mathcal {A}}\mathcal{L}_{\mathbf{a},\mathbf{M},A}V(x) \\& \quad =\inf_{(\mathbf{a},\mathbf{M},A)\in\mathcal{A}} \biggl\{ \bigl[A\mu+(x-A))\delta+C^{I} \bigr]V_{x}+\frac{1}{2}A^{2}\sigma^{2}V_{xx} \\& \qquad {}+ \int_{0}^{\infty}\bigl[V(x-y)-V(x)\bigr]\,\mathrm{d}\tilde {G}(x) \biggr\} \\& \quad =0 \end{aligned}$$

with boundary condition $V(+\infty)=0$. However, it is difficult to solve Eq. (17) even in the classical risk model with investment chance (cf. [26]). We alternatively find optimal decision policies for minimizing the upper bound, i.e., Lundberg inequality, for ruin probability in the next section.

## Minimizing upper bound of ruin probability

As it was shown in Gaier *et al.* [27], it is also reasonable to consider the “discounted wealth process” of the insurance company when we focus on minimizing ultimate ruin probability. In this case, the constant interest force can be assumed to be zero. Following this framework, in the rest of this paper, we restrict the discussion to the case that $\delta \equiv0$. Now, HJB equation (17) can be rewritten as

18 $$\begin{aligned} \inf_{(\mathbf{a},\mathbf{M},A)\in \mathcal{A}} \biggl\{ \bigl[A\mu+C^{I} \bigr]V_{x}+\frac{1}{2}A^{2}\sigma ^{2}V_{xx}+ \int_{0}^{\infty}\bigl[V(x-y)-V(x)\bigr]\,\mathrm{d} \tilde{G}(x) \biggr\} =0 \end{aligned}$$

with boundary condition $V(+\infty)=0$.

Recall that in the classical risk model and its extensions, when the claims are “small claims”, the ultimate ruin probability has an exponential upper bound of the form $\psi(x)\leq \mathrm {e}^{-Rx}$, where *R* is the Lundberg exponent. This result inspires us alternatively to find a solution to HJB equation (18) of the form

19 $$ V(x)=C_{0}\mathrm {e}^{-rx}, $$

where *r* is a constant and will be optimized later. Substituting Eq. (19) into Eq. (18) yields

20 $$\begin{aligned}& \inf_{(\mathbf{a},\mathbf{M},A)\in\mathcal {A}}\mathrm {e}^{-rx} \biggl\{ - \bigl[A \mu+C^{I} \bigr]r+\frac{1}{2}A^{2}\sigma ^{2}r^{2}+ \int_{0}^{\infty}\bigl(\mathrm {e}^{ry}-1\bigr) \,\mathrm{d}\tilde{G}(x) \biggr\} \\& \quad =\inf_{(\mathbf{a},\mathbf{M},A)\in\mathcal {A}} \biggl\{ - \bigl[A \mu+C^{I} \bigr]r+\frac{1}{2}A^{2}\sigma ^{2}r^{2}+M_{\tilde{S}(1)}(r) \biggr\} . \end{aligned}$$

### Lemma 3.1

*If we want to find a solution to the HJB equation of the form* (19), *then the optimal constant investment policy and optimal reinsurance policy are separately determined*.

### Proof

Observing Eq. (20), we may find that the variable *x* is not involved in the equation. This indicates that if one wants to maximize a “value function” of the form (19), the optimal investment and reinsurance amounts are independent of the current wealth of the insurance company. Equation (20) can be rewritten as

21 $$ \inf_{(\mathbf{a},\mathbf {M},A)\in\mathcal{A}} \biggl\{ - \biggl[A\mu r- \frac{1}{2}A^{2}\sigma ^{2}r^{2} \biggr]-C^{I} r+M_{\tilde{S}(1)}(r) \biggr\} , $$

then we find that the expression in the big brackets can be separated into two parts: one part only contains variable *A* and the other one contains variable **a**, **M**. This indicates that we can minimize the expression by finding an optimal investment strategy, say $A^{*}$, and an optimal reinsurance pair $\mathbf{a}^{*}$, $\mathbf{M}^{*}$ respectively. □

By Eq. (21), one may find that, for any given *r*, an optimal investment amount corresponding to *r* is $A^{*}(r)=\frac{\mu }{\sigma^{2}r}$. This means that if we want to minimize Lundberg inequality with exponent *r*, then the optimal investment amount is constant policy. Under the constant investment strategy, the wealth process of the insurance company is

22 $$ X_{\mathbf{a},\mathbf{M},A}(t)=A (\mu t+\sigma W_{t})+C^{I}t-\sum _{i=1}^{n}\sum_{j=1}^{N_{i}(t)} \tilde{Y}_{j}^{(i)} $$

and the infinitesimal operator of $X_{\mathbf{a},\mathbf{M},A}(t)$ is

23 $$ \bigl(A\mu+C^{I}\bigr)f_{x}+\frac{1}{2} A^{2}\sigma^{2}f_{xx}+ \int _{0}^{\infty}\bigl(f(x-y)-f(x)\bigr)\,\mathrm{d} \tilde{G}(y). $$

To proceed our discussion, we introduce the following notation $\mathbf {L}_{0}$, which defines the admissible domain of the controlled variables $(\mathbf{a},\mathbf{M},A)$:

24 $$ \mathbf{L}_{0}= \Biggl\{ (\mathbf{a},\mathbf {M},A) \Big|( \mathbf{a},\mathbf{M},A) \mbox{ satisfies Eq. } (9) \mbox{ and } \sum _{i=1}^{n} \mathbb {E}Y(a_{i},M_{i})+A \mu>0 \Biggr\} . $$

### Lemma 3.2

*The equation*

25 $$ - \bigl[A\mu+C^{I} \bigr]r+\frac{1}{2}A^{2} \sigma^{2}r^{2}+M_{\tilde{S}(1)}(r)=0 $$

*has a unique positive root if and only if* $(\mathbf{a},\mathbf {M},A)\in\mathbf{L}_{0}$.

### Proof

Denote the left-hand side of Eq. (25) by $h(r)$, then $h(0)=0$, $h'(0)=-\sum_{i=1}^{n} \mathbb {E}Y(a_{i}, M_{i})-A\mu$. Note that

26 $$ h''(r)=A^{2}\sigma^{2}+\mathbb {E}\bigl[ \exp\bigl(r\tilde{S}(t)\bigr)\tilde{S}^{2}(t) \bigr]>0, $$

thus $h(r)$ is a convex function on equation $(0,\infty)$. Thus equation $h(r)=0$ admits a positive root if only if

27 $$ h'(0)=-\sum_{i=1}^{n} \mathbb {E}Y(a_{i},M_{i})-A\mu< 0, $$

this is equal to $(\mathbf{a},\mathbf{M},A)\in\mathbf{L}_{0}$. □

To clarify the impact of **a**, **M**, *A* on the root to Eq. (25), denote the unique positive root by $R_{\mathbf {a}, \mathbf{M},A}$. Following a standard method, we have the following upper bound estimation for ultimate ruin probability.

### Theorem 3.3

*Assume that the conditions in Lemma *3.2 *are satisfied*, *then*

28 $$ \psi(x;\mathbf{a},\mathbf{M},A)=\mathbb {P}\bigl(\tau _{\mathbf{a},\mathbf{M},A}(x)< \infty \bigr) \leq \mathrm {e}^{-R_{\mathbf {a},\mathbf{M},A}x}. $$

### Proof

It is easy to verify that if $R_{\mathbf{a},\mathbf{M},A}$ is the root to Eq. (25), then $f(x)=\mathrm {e}^{-R_{\mathbf{a},\mathbf{M},A}x}$ is the solution to equation

29 $$ \bigl(A\mu+C^{I}\bigr)f_{x}+\frac{1}{2} A^{2}\sigma^{2}f_{xx}+ \int _{0}^{\infty}\bigl(f(x-y)-f(x)\bigr)\,\mathrm{d} \tilde{G}(y)=0. $$

By the martingale problem of Markov process (cf. Rogers and Williams [28]), we know that process

30 $$ \bigl(\exp\{-R_{\mathbf{a},\mathbf{M},A}X_{\mathbf {a},\mathbf{M},A}(t),\mathcal{F}_{t} \bigr)_{t\geq0} $$

is a martingale, and by an optional sampling theorem of martingale, we have

$$\begin{aligned}& \mathrm {e}^{-R_{\mathbf{a},\mathbf{M},A}x} \\& \quad =\mathbb {E}\bigl[\exp\bigl\{ -R_{\mathbf{a},\mathbf{M},A}X_{\mathbf {a},\mathbf{M},A}\bigl( \tau_{\mathbf{a},\mathbf{M},A}(x)\wedge t\bigr)\bigr\} \bigr] \\& \quad =\mathbb {E}\bigl[\exp\bigl\{ -R_{\mathbf{a},\mathbf{M},A}X_{\mathbf {a},\mathbf{M},A}(t)\bigr\} 1_{(\tau_{\mathbf{a},\mathbf{M},A}(x)> t)} \bigr] \\& \qquad {}+\mathbb {E}\bigl[\exp\bigl\{ -R_{\mathbf{a},\mathbf{M},A}X_{\mathbf {a},\mathbf{M},A}\bigl( \tau_{\mathbf{a},\mathbf{M},A}(x)\bigr)\bigr\} 1_{(\tau _{\mathbf{a},\mathbf{M},A}(x)\leq t)} \bigr] \\& \quad \geq \mathbb {E}\bigl[\exp\bigl\{ -R_{\mathbf{a},\mathbf{M},A}X_{\mathbf {a},\mathbf{M},A}\bigl( \tau_{\mathbf{a},\mathbf{M},A}(x)\bigr)\bigr\} 1_{(\tau _{\mathbf{a},\mathbf{M},A}(x)\leq t)} \bigr] \\& \quad =\mathbb {E}\bigl[\exp\bigl\{ -R_{\mathbf{a},\mathbf{M},A}X_{\mathbf {a},\mathbf{M},A}\bigl( \tau_{\mathbf{a},\mathbf{M},A}(x)\bigr)\bigr\} |\tau _{\mathbf{a},\mathbf{M},A}(x)\leq t \bigr]\mathbb {P}\bigl( \tau_{\mathbf {a},\mathbf{M},A}(x)\leq t\bigr). \end{aligned}$$

Thus

31 $$\begin{aligned}& \mathbb {P}\bigl(\tau_{\mathbf{a},\mathbf{M},A}(x)\leq t\bigr) \\& \quad \leq \frac{\mathrm {e}^{-R_{\mathbf{a},\mathbf{M},A}x}}{ \mathbb {E}[\exp\{-R_{\mathbf{a},\mathbf{M},A}X_{\mathbf{a},\mathbf {M},A}(\tau_{\mathbf{a},\mathbf{M},A}(x))\} |\tau_{\mathbf {a},\mathbf{M},A}(x) \leq t ]}. \end{aligned}$$

Note that $\tau_{\mathbf{a},\mathbf{M},A}(x)$ is the ruin time of the insurer with policies **a**, **M**, *A*, thus $X_{\mathbf {a},\mathbf{M},A}(\tau_{\mathbf{a},\mathbf{M},A}(x))\leq0$, this means the denominator in Eq. (31) is greater than or equal to 1. This leads to

32 $$\begin{aligned} \mathbb {P}\bigl(\tau_{\mathbf{a},\mathbf{M},A}(x)\leq t\bigr)\leq \mathrm {e}^{-R_{\mathbf{a},\mathbf{M},A}x}. \end{aligned}$$

The proof is completed by letting $t\rightarrow\infty$. □

### Remark 3

Lemma 3.2 seems similar to Lemma 1 of [22]. However, the sufficient and necessary conditions such that the Lundberg coefficient exists in [22] is the net profit condition (8). By condition (27) we know that, because of the investment chance, the insurance company can derive an exponential upper bound estimation even if the net profit condition (8) does not hold. This indicates that investment offers the insurance company more chance to control its exposure. Then, the remaining problem is how the insurance company should settle the investment position to obtain the minimal upper bound of ruin probability.

Now we want to find optimal **a**, **M**, *A* such that $R_{\mathbf{a},\mathbf{M},A}$ attains its maximum and consequently the minimal upper bound for ultimate ruin probability. By Lemma 3.1, the optimal investment and optimal reinsurance policies are separately determined.

1. *Optimal investment policy* Note that by dynamic programming, this is equal to finding the optimizer of Eq. (20). Denote
  33 $$ h(R):=- \biggl[A_{t}\mu R-\frac{1}{2}A_{t}^{2} \sigma^{2}R^{2} \biggr]-C^{I} R+M_{\tilde{S}(1)}(R), $$
  then
  34 $$ A^{*}=\frac{\mu}{\sigma^{2} R} $$
  is the maximizer of $h(R)$. Substitute (34) into Eq. (25), the maximum $R^{*}$ should be the solution to equation
  35 $$ M_{\tilde{S}(1)}(R)-C^{I}R-\frac{1}{2} \frac{\mu^{2}}{\sigma^{2}}=0. $$
  Solving Eq. (35) we have an optimal investment amount immediately. To proceed our discussion, let $H_{\mathbf{a},\mathbf{M},A^{*}}(R):=M_{\tilde{S}(1)}(R)-C^{I}R$ and $a_{i}'=\frac{\theta_{i}^{R}-\theta_{i}}{\theta_{i}^{R}}$.
2. *Optimal reinsurance policy* Suppose that the optimal investment policy is determined, then the optimal reinsurance strategy shall maximize the $R_{\mathbf{a},\mathbf{M},A^{*}}$ w.r.t $(\mathbf {a},\mathbf{M})$. This is equivalent to determining the optimal quota-share retention levels $\mathbf{a^{*}}= (a^{*}_{1},\ldots,a^{*}_{n})$, and excess of loss retention limits $\mathbf {M^{*}}=(M^{*}_{1},\ldots,M^{*}_{n})$ such that $R_{\mathbf{a},\mathbf {M},A^{*}}$ attains maximum. By Theorem 3.1, the optimal investment amount and optimal reinsurance policies are separately determined, the properties obtained in [22] can be applied directly. In the rest of this paper, we focus on the impact of investment chance on the upper bound of ruin probability by numerical examples.

## Numerical examples

By previous results, we know that the optimal reinsurance policies and optimal investment amounts are separately determined, and the investment chance implies that the adjustment coefficient in our risk model is less than that in the model of [22]. However, since we have no analytical expression for the solution to the Lundberg equation, we cannot compare the minimal upper bound in our risk model and the one in [22] directly. In this section, we will illustrate some comparative results by numerical examples. For this goal, we choose the same surplus process parameters as those in [22]. To illustrate the effect of investment chance, we compare the Lundberg exponent in [22] and the one in our model. Denote the maximum Lundberg exponent in [22] and the one in our model by $R_{H}^{*}$ and $R^{*}$, respectively.

### Example 1

Let $Y^{(1)}$ and $Y^{(2)}$ be exponentially distributed with means $\mu_{1} = \mu_{2}=1$, which are typical “small claim”. Let the parameters of the financial market be $\mu=0.1$, $\sigma=0.05$, and thus the market price of the market is 2. For any given $(\theta_{1}, \theta_{1}^{R}1)$ and $(\theta_{2}, \theta_{2}^{R})$, by calculating the optimal excess of loss retention levels $(M_{1}^{*},M_{2}^{*})$ with $(\alpha_{11}, \alpha_{12})$ and $(\alpha_{21}, \alpha_{22})$, we have the correspondingly upper bound of ruin probability under different structure.

The numerical results in Tables 1 and 2 show that when the correlation coefficient *ρ* increases, both the Lundberg exponent in [22] and the one in this paper decrease. This means that the risk exposure of the insurance company increases because the upper bound of ruin probability is determined by the Lundberg exponent. On the other hand, due to the existence of the investment chance, the Lundberg exponent in this paper is less than the one in [22], which means that the extra investment chance can really decrease the risk exposure of the insurance company. *However, one should note that when* *ρ* *is increasing, the decreasing effect caused by the investment chance is decreasing (the difference between* $R_{H}^{*}$ *and* $R^{*}$ *is decreasing). This means that when the business correlations of the insurance company are increasing, the affect of investment is decreasing. This result coincides with the routine risk management rules in the insurance company: control system risk is more important than control market risk.*

**Table 1:** Comparison of Lundberg exponent: exponential case. $(\theta _{1}, \theta_{1}^{R})=(\theta_{2}, \theta_{2}^{R})= (0.2, 0.4)$, values of Lundberg exponent and upper bound of ruin probability with different dependence parameters for Hu’s model and our model

$(\alpha_{11},\alpha_{12})$	(0,1)	(0.2,0,8)	(0.4,0.6)	(0.6,0.4)	(0.8,0.2)	(1,0)
$(\alpha_{21},\alpha_{22})$	(1,0)	(0.8,0.2)	(0.6,0.4)	(0.4,0.6)	(0.2,0.8)	(0,1)
$(\lambda_{1},\lambda_{2})$	(4,2)	(3.6,2.4)	(3.2,2.8)	(2.8,3.2)	(2.4,3.6)	(2,4)
*ρ*	0	0.111111	0.160714	0.160714	0.111111	0
$M^{*}_{1}$	1.48575	1.595619	1.602443	1.542483	1.47389	1.48575
$M^{*}_{2}$	1.48575	1.47389	1.542483	1.602443	1.595619	1.48575
$R_{H}^{*} $	0.226466	0.184908	0.169814	0.169814	0.184908	0.226466
$e^{-10R_{H}^{*}}$	0.1038	0.1573	0.1830	0.1830	0.1573	0.1038
$R^{*} $	0.231466	0.194724	0.193215	0.173001	0.194032	0.231298
$e^{-10R^{*}}$	0.0988	0.1427	0.1769	0.1773	0.1437	0.0099

**Table 2:** Comparison of Lundberg exponent: exponential case. $(\theta _{1}, \theta_{1}^{R})=(0.2, 0.4)$ and $(\theta_{2}, \theta_{2}^{R})= (0.25, 0.5)$, values of Lundberg exponent and upper bound of ruin probability with different dependence parameters for Hu’s model and our model

$(\alpha_{11},\alpha_{12})$	(0,1)	(0.2,0,8)	(0.4,0.6)	(0.6,0.4)	(0.8,0.2)	(1,0)
$(\alpha_{21},\alpha_{22})$	(1,0)	(0.8,0.2)	(0.6,0.4)	(0.4,0.6)	(0.2,0.8)	(0,1)
$(\lambda_{1},\lambda_{2})$	(4,2)	(3.6,2.4)	(3.2,2.8)	(2.8,3.2)	(2.4,3.6)	(2,4)
*ρ*	0	0.111111	0.160714	0.160714	0.111111	0
$M^{*}_{1}$	1.374693	1.426397	1.382661	1.292643	1.22716	1.286787
$M^{*}_{2} $	1.65657	1.679524	1.741289	1.766061	1.70904	1.55064
$R_{H}^{*} $	0.244762	0.201829	0.188391	0.191707	0.211816	0.261482
$e^{-10R_{H}^{*}}$	0.0865	0.1329	0.1520	0.1470	0.1203	0.0732
$R^{*} $	0.273025	0.228736	0.203113	0.202304	0.231029	0.271354
$e^{-10R^{*}}$	0.0652	0.1015	0.1312	0.1323	0.0992	0.0663

### Example 2

Let the distributions of $Y^{(1)}$ and $Y^{(2)}$ be Pareto $(2,1)$, with common c.d.f. $F(y) =1- 1/(1 + y)^{2}, y> 0$, then $\mathbb {E}Y^{(1)}=\mathbb {E}Y^{(2)} = 1$. Although in this example the means of individual claim amount are equal to those in Example 1, the potential losses are larger since Pareto distribution is heavy-tailed. Other parameters, such as those of the financial market and those of the dependent structure, are the same as in Example 1. The calculating results are listed in Tables 3 and 4. Usually, since $Y^{(1)}$ and $Y^{(2)}$ have heavy tails, their moment generating function does not exist. However, due to the reinsurance policy settled in this paper, the tail losses of $Y^{(1)}$ and $Y^{(2)}$ are truncated, thus we can calculate the corresponding $M^{*}_{1}$ and $M^{*}_{2}$. [22] made some conclusions based on this kind of distribution. We may find that, although the individual claims are heavy-tailed, the investment chance can really increase the Lundberg exponent and thus decrease the upper bound of ruin probability. But the gap between $R_{H}^{*}$ and $R^{*}$ is very tiny. This means that, in the situation of heavy tail, especially accompanied with high correlated business, the investment cannot decrease the upper bound of ruin probability significantly. *Thus, when the claims are heavy-tailed, reinsurance plays a more important role for the insurance company than the investment. However, in a heavy-tailed case, most of the large losses are transit to the reinsurance company with a relative big probability (heavy-tailed property). From practical review, this leads to the conclusion that an optimal investment policy for the insurance company may not be accepted by the reinsurance company when we take the ruin probability as the measure of risk exposure.*

**Table 3:** Comparison of Lundberg exponent: Pareto distribution. $(\theta_{1}, \theta_{1}^{R})=(\theta_{2}, \theta_{2}^{R})= (0.2, 0.4)$, values of Lundberg exponent and upper bound of ruin probability with different dependence parameters for Hu’s model and our model

$(\alpha_{11},\alpha_{12})$	(0,1)	(0.2,0,8)	(0.4,0.6)	(0.6,0.4)	(0.8,0.2)	(1,0)
$(\alpha_{21},\alpha_{22})$	(1,0)	(0.8,0.2)	(0.6,0.4)	(0.4,0.6)	(0.2,0.8)	(0,1)
$(\lambda_{1},\lambda_{2})$	(4,2)	(3.6,2.4)	(3.2,2.8)	(2.8,3.2)	(2.4,3.6)	(2,4)
*ρ*	0	0.111111	0.160714	0.160714	0.111111	0
$M^{*}_{1}$	2.325091	2.485092	2.521622	2.470896	2.380495	2.325091
$M^{*}_{2} $	2.325091	2.380495	2.470896	2.521622	2.485092	2.325091
$R_{H}^{*} $	0.144713	0.125136	0.117427	0.117427	0.125136	0.144713
$e^{-10R_{H}^{*}} $	0.2352	0.2861	0.3090	0.3090	0.2861	0.2352
$R^{*} $	0.160045	0.132486	0.120324	0.120011	0.127033	0.145129
$e^{-10R^{*}}$	0.2018	0.2658	0.3002	0.3012	0.2807	0.2343

**Table 4:** Comparison of Lundberg exponent: exponential case. $(\theta _{1}, \theta_{1}^{R})=(0.2, 0.4)$ and $(\theta_{2}, \theta_{2}^{R})= (0.25, 0.5)$, values of Lundberg exponent and upper bound of ruin probability with different dependence parameters for Hu’s model and our model

$(\alpha_{11},\alpha_{12})$	(0,1)	(0.2,0,8)	(0.4,0.6)	(0.6,0.4)	(0.8,0.2)	(1,0)
$(\alpha_{21},\alpha_{22})$	(1,0)	(0.8,0.2)	(0.6,0.4)	(0.4,0.6)	(0.2,0.8)	(0,1)
$(\lambda_{1},\lambda_{2})$	(4,2)	(3.6,2.4)	(3.2,2.8)	(2.8,3.2)	(2.4,3.6)	(2,4)
*ρ*	0	0.111111	0.160714	0.160714	0.111111	0
$M^{*}_{1} $	2.151698	2.245443	2.223645	2.134013	2.03478	2.012961
$M^{*}_{2} $	2.592899	2.663988	2.731697	2.735907	2.641812	2.4257
$R^{*}$	0.1564	0.1368	0.1303	0.1323	0.1431	0.1672
$e^{-10R^{*}} $	0.209349	0.254557	0.271713	0.266337	0.239386	0.18796
$R^{*} $	0.162025	0.139998	0.135537	0.137071	0.148071	0.169951
$e^{-10R^{*}}$	0.1978	0.2466	0.2579	0.2539	0.2275	0.1828
